# Antioxidant Formulae, Shengmai San, and LingGuiZhuGanTang, Prevent MPTP Induced Brain Dysfunction and Oxidative Damage in Mice

**DOI:** 10.1155/2015/584018

**Published:** 2015-11-05

**Authors:** Vijayasree Vayalanellore Giridharan, Rajarajan Amirthalingam Thandavarayan, Tetsuya Konishi

**Affiliations:** ^1^J.K.K. Nattraja College of Pharmacy, Komarapalayam, Tamil Nadu 638 183, India; ^2^Niigata University of Pharmacy & Applied Life Sciences, Niigata 956 8603, Japan; ^3^Department of Cardiovascular Sciences, Houston Methodist Research Institute, Houston, TX 77030, USA; ^4^Changchun University of Chinese Medicine, Bosuo Road No. 1035, Jingyue Economic Development District, Changchun 130117, China

## Abstract

The present study was designed to evaluate the preventive effect of antioxidative traditional oriental medicine formulae, Shengmai San (SMS) and LingGuiZhuGanTang (LGZGT), against 1-methyl-4-phenyl-1,2,3,6-tetrahydropyridine (MPTP) (i.p 30 mg·kg^−1^ for 5 consecutive days) induced neurotoxicity. In *in vitro* antioxidant assays measured with Trolox and butyl hydroxyl toluene as reference antioxidant revealed that SMS has higher scavenging potential against hydroxyl radical than superoxide anion radical, but LGZGT was the reverse. The neuroprotective effect of SMS and LGZGT against MPTP was evaluated in mice by behavioral, biochemical, and immunohistochemical studies. In the behavioral study, both SMS and LGZGT significantly reversed the locomotive impairment induced by MPTP. Simultaneously, both formulae significantly prevented the MPTP induced dopaminergic neuron loss assessed by tyrosine hydroxylase in the midbrain. Both SMS and LGZGT significantly attenuated the elevated lipid peroxidation and protein carbonyls levels by MPTP. The DNA damage induced by MPTP was also prevented by both formulae. Although a little difference in the protective functions was observed between the two formulae, such as in DNA damage and behavioral studies, the results indicate that both SMS and LGZGT with antioxidant property act as a good candidate applicable for the antioxidant based complementary therapies of neurodegenerative diseases.

## 1. Introduction

Parkinson's disease (PD) is a common adult-onset neurodegenerative disorder and over the period of time patients see their disability gradually growing and their quality of life declining [1]. The cause of idiopathic PD is still unknown, but complex features including aging, environmental factors, oxidative stress, neuroinflammation, and genetic factors are involved in the development of the disease [2, 3]. Reactive oxygen species (ROS) and free radicals are implicated as the species playing critical roles in etiology and pathogenesis of age related neurodegenerative diseases, including PD [4–6].

The 1-methyl-4-phenyl-1,2,3,6-tetrahydropyridine (MPTP) administration in rodents is one of the models that mimics the PD phenotype. Numerous studies have shown that the administration of MPTP causes neuronal cell defect in the* substantia nigra pars compacta* (SNpc) [4, 7] and decline in locomotive activity and rearing frequencies [8, 9]. ROS produced in the physiological metabolisms of MPTP is the major factor causing these pathological conditions; hence, overproduction of ROS causes oxidative stress to cellular critical molecules such as DNA, lipids, and proteins leading to many disorders [10]. Therefore, the antioxidant protection might be a primary strategy for treating or preventing PD.

On the other side, current approaches to the treatment of PD suffer from the “wearing off” effect. A majority of patients treated with levodopa experience motor fluctuations, dyskinesia, or other complications after a period of time [11, 12]. So, it is mandatory to discover alternate pharmacological agents for the treatment of PD that will be effective either alone or in combination with presently used drugs to achieve maximum benefit with least side effects.

Traditional oriental medicines such as traditional Chinese medicines (TCM) and Kampo medicines are the composite formula of herbs and have been used historically for treating wide variety of disorders with an aim of enhancing physiological homeostatic potential. The brain is playing an important role in the physiological homeostasis and is also known as a weak organ against oxidative stress. Further, the pathogenic process of degenerative disorders is complex and involves many factors [1]. The antioxidant based composite therapy will thus be a promising approach against neurodegenerative diseases. Since different formulae are often used for the same disease condition and different pathological condition is treated by the same formula in the traditional oriental medicines, the oxidative stress is implicated as the common and basic target of them [13]. In this sense, the antioxidant traditional medicines are an attractive target of study as the model of antioxidant composite formula applicable for degenerative brain damage.

In the present study, we focused on the protective potential of two TCM formulae with different property against MPTP induced brain damages. Shengmai San (SMS), comprising three crude drug components, Radix Ginseng (*Panax ginseng*; Araliaceae), Radix Ophiopogonis (*Ophiopogon japonicus*; Liliaceae), and Fructus Schisandrae (*Schisandra chinensis*; Schisandraceae), has been traditionally used for treating heart disease [14, 15]. LingGuiZhuGanTang (LGZGT), on the other hand, is composed of four crude drug components, Cinnamon twig (*Cinnamomum cassia *Presl.; Lauraceae), Atractylodis rhizoma (*Atractylodes macrocephala* Koidz.; Compositae), Glycyrrhizae radix (*Glycyrrhiza uralensis* Fisch.; Leguminosae), and one fungi, Hoelen (*Poria cocos* (Schw.) Wolf Polyporaceae) which is often used for diseases related to edema, chronic bronchitis, heart failure, and chronic nephritis [16]. The antioxidant and neuroprotective potentials of these formulae were studied in MPTP induced PD model mice using behavioral, biochemical, and immunohistochemical studies to assess the beneficial use of these antioxidant composite formulae in complimentary or integrated therapy of degenerated brain disorders.

## 2. Materials and Methods

### 2.1. Animals

Male C57BL/6 mice (40 animals; 12–15 weeks old; 25–30 g) were purchased from SLC Inc., Japan. The animals were randomly housed in groups of five in polypropylene cages with wood shavings as bedding and maintained in a temperature controlled room (22 ± 2°C) on a 12 h light dark cycle (lights on at 7:00 am). The animals had free access to water and food throughout the experiment and were used after a 1 week adaptation period. The studies were carried out in accordance with the guidelines of the Committee on the Care and Use of Laboratory Animals, Niigata University of Pharmacy and Applied life Sciences, Japan.

### 2.2. Materials

SMS and LGZGT are commercially available ready to eat granules and were generously provided by Iskura Co., Ltd., Japan, and by Kotaro Co., Ltd., Japan, respectively. 5,5 Dimethyl 1 pyrroline N oxide (DMPO) was purchased from Labotec Co., Ltd., Japan. 6-Hydroxy-2,5,7,8 tetramethylchroman-2-carboxylic acid (Trolox) was purchased from Aldrich Chem. Co. Anti-dinitrophenyl (DNP) IgG developed in rabbit, and 2,4-dinitrophenylhydrazine (DNPH) and MPTP were from Sigma Co., Ltd., USA. Rabbit antimouse IgG conjugated to horseradish peroxidase (HRP) was obtained from Zymed, USA. 3,3′,5,5′ Tetramethylbenzidine (TMB) was obtained from Bio-Rad Lab., USA. All other chemicals were purchased from Wako Pure chemicals Industries Co., Ltd., Japan.

### 2.3. Experimental Protocol

In this study, the mice were randomly divided into 4 (*n* = 10) groups: group 1: normal control + vehicle (water treatment), group 2: MPTP treatment alone (30 mg·kg^−1^ i.p for 5 consecutive days), group 3: SMS + MPTP treatment, and group 4: LGZGT + MPTP treatment. The animals received the SMS and/or LGZGT treatment on day 1 to day 10. SMS and/or LGZGT granules were suspended in water and administered intragastrically at the dose of 8 g·kg^−1^ to the respective groups. The dose was selected from our previous studies [17, 18]. The MPTP treatment was given to the same animals from day 6 to day 10. On day 11, the animals were subjected to behavioral study at 24 h after last injection of MPTP and undergone biochemical assay (*n* = 5) and immunohistochemical study (*n* = 5).

### 2.4. Hydroxyl Radical Scavenging Activity Measurement* In Vitro*

Fenton reaction was used for the production of HO^∙^ and its scavenging activity was determined as reported previously [19]. DMPO used as a spin trap, in the X-Band Microwave Unit, JEOL JES-TE 200 ESR spectrometer for the detection of the electron spin resonance (ESR) spectra of DMPO-OH with the following settings: microwave power; 8 mW, microwave frequency; 9.41 GHz, modulation amplitude; 0.1 mT, time constant; 0.03 s, sweep time; 30 s, center fields; 340.0/335.4 mT.

### 2.5. Superoxide Scavenging Activity Measurement* In Vitro*

Hypoxanthine and xanthine oxidase was used for the generation of superoxide and its scavenging activity was measured according to the methods reported previously [20]. DMPO was used as a spin trap. The ESR settings for DMPO-OOH was microwave power; 8 mW, microwave frequency; 9.41 GHz, modulation amplitude; 0.1 mT, time constant; 0.03 s, sweep time; 30 s, center fields; 340.0/335.4 mT.

### 2.6. DPPH Radical Scavenging Assay* In Vitro*

DPPH radical scavenging activity of SMS and LGZGT was determined according to the method of Shin et al. [21]. Briefly, 1 mg·mL^−1^ of SMS and/or LGZGT was added to 3 mL of DPPH (0.15 mM) solution. The mixtures were shaken vigorously and left to stand at room temperature in the dark for 30 min. The change of DPPH concentration was monitored at 517 nm wavelength.

### 2.7. Open Field Test

The test was used to determine motor-function alterations of animals. Open field test was done on a rectangular box (40 × 50 × 63 cm) and its floor was divided into 20 (10 × 10) small rectangles. The mice were placed in one of the corners of the rectangular box and the number of crossings between the small rectangles and the number of times the animals stood using their hind paws also called as rearing were determined. Further, the immobility time was measured by the lack of movement in seconds during testing and latency to start the movement was determined by the time taken for the animal to leave the first rectangle. All these parameters were determined by hand operated counters for the duration of 5 min [22]. The rectangular box was washed with a 5% alcohol solution before placing each animal.

### 2.8. Pole Test

The pole test for bradykinesia was conducted by using a modification of the reported method [23]. In a rough surfaced pole the mouse was placed upward (10 mm diameter and 55 cm height), and *T*_turn_ (time from the beginning of movement until the mouse turns completely downward) and *T*_LA_ (time until it arrives at floor) were measured. The elongation of these parameters is considered to reflect bradykinesia. This test was performed five times successively for each mouse [24].

### 2.9. Tissue Homogenate Preparation

The animals were sacrificed after behavioral study, and the whole brain was removed. The midbrain and cortex were dissected and then chilled in ice-cold saline. After washing with 0.85% NaCl, the tissue was suspended in cold 0.05 M phosphate buffer containing 1.15% (w/v) KCl (9 mL·1 g^−1^ wet tissue) and then homogenized at 4°C using an ULTRA TRAX homogenizer. The protein concentration of the homogenate was determined by BCA protein assay using bovine serum albumin as a standard.

### 2.10. Measurement of Lipid Peroxidation

The lipid peroxidation product in brain homogenates was assayed by measurement of the malondialdehyde (MDA) concentration according to the method elsewhere [25]. Brain homogenates (0.4 mg of protein·mL^−1^) were mixed with 1 mL of 1% thiobarbituric acid in 50 mM sodium hydroxide and 1 mL of 2.8% trichloroacetic acid, and the concentration of MDA was measured. The MDA concentration is expressed as nanomoles per milligram of protein.

### 2.11. Measurement of Protein Carbonyl Content

A sensitive enzyme linked immunosorbent assay (ELISA) was employed to determine protein carbonyl content as previously reported [26]. Briefly, the supernatants of tissue homogenate obtained after centrifugation were incubated with 1% streptomycin sulfate. The homogenate (1 mg as protein) was suspended in PBS (phosphate buffer) and allowed to react with 10 mM DNPH in 2 N HCl in darkness at room temperature for 1 h. The protein was precipitated with 20% trichloro acetic acid and then solubilized in PBST (PBS + tween 20) and was placed in 96-well plate and incubated overnight at 4°C. The samples were incubated 4 h with a primary antibody (anti-DNP rabbit IgG) at 37°C and washed with PBST, then reacted with a secondary antibody (anti-mouse rabbit IgG HRP conjugate) for 1 h. Peroxidase reactions were performed with the addition of TMB for an hour and stopped with the addition of sulphuric acid. Absorbance was measured at 450 nm using a Bio-Rad model 550 microplate reader.

### 2.12. Alkaline Single-Cell Gel Electrophoresis Assay

DNA damage was detected by alkaline single-cell gel electrophoresis assay (Comet assay) [27, 28]. Briefly, brain tissue was removed immediately after sacrificing the mice and washed with chilled PBS. The tissue was dissociated into cells using a cell dissociation sieve-tissue grinder kit under ice-cold conditions and processed for the Comet assay. Approximately, 1 × 10^5^ cells were mixed with 1.5 mL of 0.8% low melting agarose solution prepared in 0.9% saline at 38°C and poured onto fully frosted microscope slides. After solidification, slides were immersed in lysis buffer (2.5 M NaCl, 100 mMNa_2_-EDTA, 1% Triton X-100, and 10% dimethyl sulfoxide) for 1 h at 4°C and electrophoresed in alkaline buffer (300 mM NaOH, 1 mM Na_2_-EDTA, pH 13) using 25 V, 400 mA for 30 min. After electrophoresis, the slides were washed gently in a neutralizing buffer (0.4 M Tris-HCl, pH 7.5) to remove the alkaline buffer and detergent and stored on wet tissue paper in a closed plastic box at 4°C until observation. The slides were stained with SYBR Green II, and at least 50 cells were captured per slide at ×200 magnification usinga fluorescence microscope (Olympus (BH2-RFCA), Japan). The digital imaging CASP software (http://casp.sourceforge.net/) was used to measure the indexes of DNA damage. Tail length and tail moment were selected as the parameters to quantify DNA damage.

### 2.13. Tyrosine Hydroxylase (TH) Immunohistochemistry

After the behavioral study, the mice were anesthetized with ketamine and were intracardially perfused with saline and the entire mesencephalon was removed and fixed using 4% paraformaldehyde. The paraffin sections (16 *μ*m thick) encompassing were prepared for TH immunohistochemical staining. Following blocking with 8% skimmed milk for 40 min at room temperature, sections were incubated with rabbit anti-TH antibody (diluted 1 : 200) at 4°C overnight. The avidin-biotin immunoperoxidase method was used for the detection of positive reaction with the Vectastain ABC Kit (Vector Laboratories, Burlingame, CA) according to manufacturer's instructions. The bound peroxidase was stained with 0.05%, 3.3′-diaminobenzidine with 0.03% hydrogen peroxide in Tris-HCl buffer, pH 7.6, followed by counterstaining with methyl green [29]. The number of TH-positive neurons in 4 sections of the SN in each mouse was counted under light microscopy at a magnification of ×400. The average of right and left neuronal cells (4 sections) was regarded as the positive cells number of each animal. The mean number of TH-positive neurons for each representative mesencephalic section was calculated three times under the final magnification of ×400.

### 2.14. Statistical Analysis

All the results were expressed as mean ± standard error (SE). The data were analyzed using one-way ANOVA followed by Tukey's* post hoc* test and Student's (unpaired) *t*-test. *P* < 0.05 was considered as statistically significant.

## 3. Results

### 3.1. *In Vitro* Antioxidant Potential of TCM Formulae

The formation of superoxide (DMPO-OOH) and hydroxyl radical (DMPO-OH) spin adducts were inhibited by the addition of SMS and/or LGZGT and the IC_50_ was evaluated. The results showed that both SMS and LGZGT showed comparable antioxidant potential with Trolox and butyl hydroxyl toluene (BHT) measured as reference antioxidant, but both formulae showed slight different specificity towards radical species. LGZGT showed rather stronger superoxide radical scavenging activity and SMS, on the other hand, showed potent scavenging activity against hydroxyl radicals. DPPH radical scavenging potential was almost the same for both formulae. The IC_50_ values of SMS, LGZGT and reference antioxidants are summarized in Table 1.

### 3.2. Effect of TCM Formulae on Neurobehavioral Study

**Figure 1:**
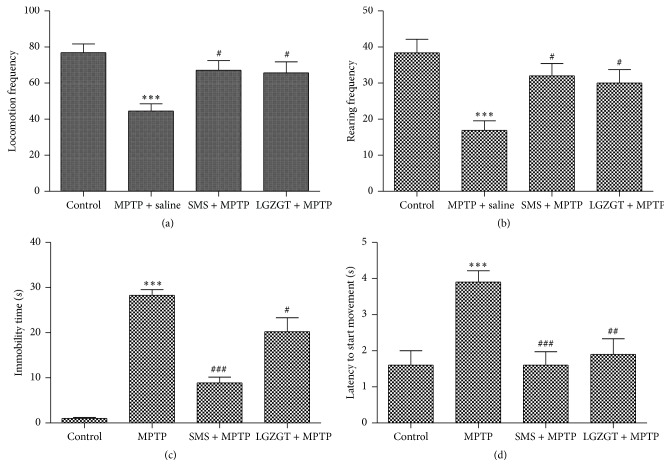
Effects of SMS and LGZGT on general motor activity impairment in MPTP treated mice (open field test): (a) locomotion frequency; (b) rearing frequency; (c) immobility time; (d) latency to start moment. Each value represents the mean ± SE. *N* = 10, ^*∗∗∗*^ *P* < 0.001: significantly different from control group. ^###^ *P* < 0.001, ^##^ *P* < 0.01, and ^#^ *P* < 0.05: significantly different from the MPTP only treated group.

The preventive effect of SMS and/or LGZGT on MPTP induced locomotive dysfunction and dyskinesia was studied by open field test and pole test, respectively. As was observed elsewhere [8, 9], the MPTP group exhibited a significant decrease in locomotion, rearing frequencies (*P* < 0.001, Figures 1(a) and 1(b)) as compared to control group in the open field test. However, both SMS + MPTP and LGZGT + MPTP groups showed a significant recovery in the locomotion and rearing frequencies (*P* < 0.05) compared to the MPTP group. In addition, SMS (*P* < 0.001) and LGZGT (*P* < 0.001) groups showed a significant decrease in immobility time and latency to start the movement when compared to the MPTP group (Figures 1(c) and 1(d)).

**Figure 2:**
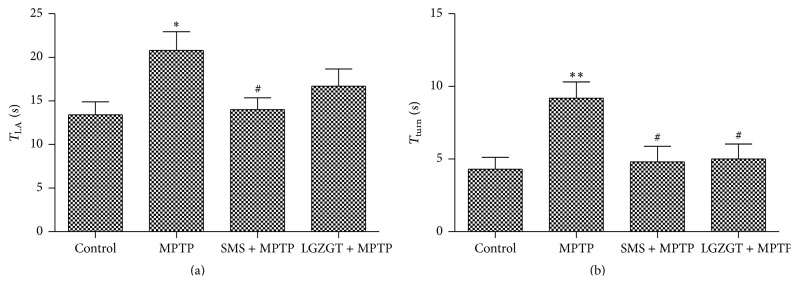
Effects of SMS and LGZGT on MPTP induced dyskinesia assessed by pole test. The mouse was placed head upward near the top of a rough surfaced pole. The time needed for the animal to turn completely downward (*T*_turn_) (a) and to reach the floor (*T*_LA_) (b) was determined. Each value represents the mean ± SE. *N* = 10, ^*∗∗*^ *P* < 0.01, ^*∗*^ *P* < 0.05: significantly different from control group. ^#^ *P* < 0.05: significantly different from the MPTP only treated group.

The protective effect of SMS and LGZGT against MPTP induced dyskinesia was examined by the pole test and the results shown in Figure 2. The MPTP group exhibited increase in the time taken in both *T*_turn_ (*P* < 0.01) and *T*_LA_ (*P* < 0.05) as compared to control group. Treatment with SMS significantly (*P* < 0.05) reduced the time taken both in *T*_LA_ and *T*_turn_ as compared to MPTP treated group (Figures 2(a) and 2(b)). LGZGT also reduced *T*_LA_ and *T*_turn_, but only the effect was significant in *T*_turn_ (Figures 2(a) and 2(b)).

### 3.3. Effect of TCM Formulae on Oxidative Stress in Brain

**Figure 3:**
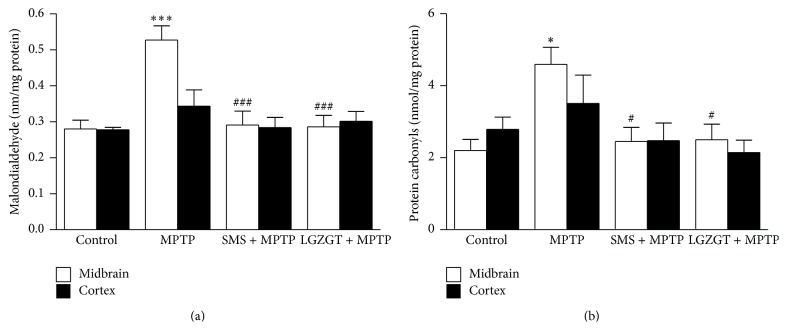
Prevention of MPTP induced oxidative stress in brain by SMS and LGZGT. Lipid peroxidation (a) and protein carbonyls (b) in tissue homogenates of midbrain and cortex were quantified as described in Section 2. Data represent nmolmg^−1^ protein and mean ± SE. *N* = 5, ^*∗∗∗*^ *P* < 0.001, ^*∗*^ *P* < 0.05, significantly differ from control group. ^###^ *P* < 0.001, ^#^ *P* < 0.05, significantly differs from the MPTP only treated group.

Oxidative damage production in the brain of MPTP treated mice was assessed by measuring MDA and protein carbonyls formation as lipid peroxidation and protein oxidation markers, respectively. On the day 11, 5 animals from each group were undergone biochemical estimation after behavioral study. The mice treated with MPTP markedly increased the MDA (*P* < 0.001)and protein carbonyls (*P* < 0.05) levels in the midbrain (Figures 3(a) and 3(b)). However, a little regional diversity was observed between MDA and protein carbonyl formation in the brain such that in midbrain; both MDA and carbonyl formations were significantly increased, but, in cortex, only carbonyls formation was increased as in the midbrain although the increase in cortex was not statistically significant. Treatment with SMS and/or LGZGT suppressed both MDA (*P* < 0.001) and carbonyls formations (*P* < 0.05) to the levels comparable to these of control mice.

### 3.4. Protective Effect of TCM on DNA Damages Assed by Comet Assay

**Figure 4:**
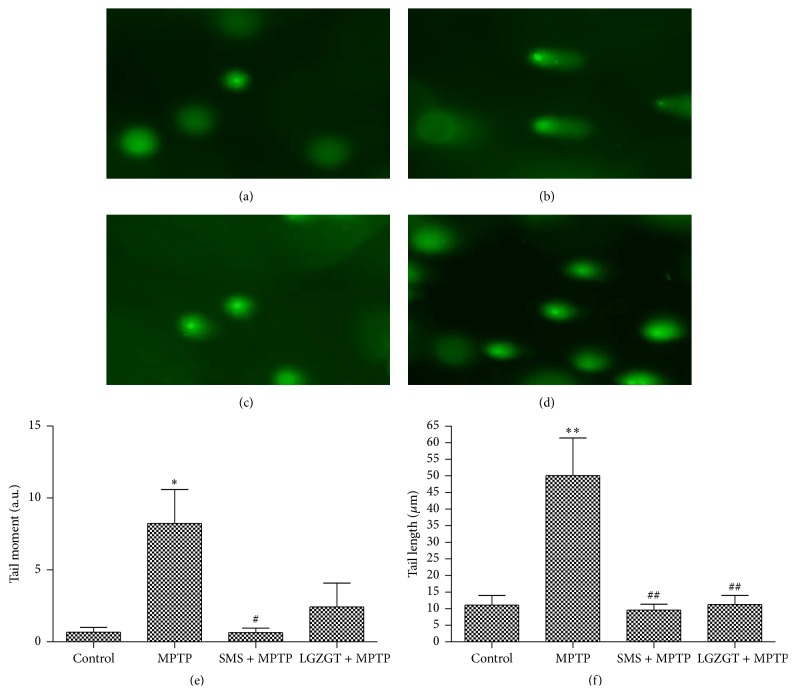
Prevention of MPTP induced DNA damage by SMS and LGZGT using Comet assay. DNA damage was determined by alkaline single-cell gel electrophoresis assay (Comet assay) in brain tissue, (a) control, (b) MPTP + saline, (c) SMS + MPTP, and (d) LGZGT + MPTP. Typical figures are at 200x original magnification. (e) Tail moment, (f) tail length. Values are mean ± SE. *N* = 4, ^*∗∗*^ *P* < 0.01, ^*∗*^ *P* < 0.05: significantly different from control group. ^##^ *P* < 0.01, ^#^ *P* < 0.05: significantly different from the MPTP only treated group.

DNA damage was further assessed for the brain cells using Comet assay as another marker of brain oxidative damage caused by MPTP. The length of Comet tail is proportional to the extent of DNA strand breaks. The DNA damage measured in terms of tail length (*P* < 0.01), and tail moment (*P* < 0.05) was significantly increased in the mice treated with MPTP as compared to control. The MPTP induced DNA damage was significantly decreased in both SMS (*P* < 0.01) and LGZGT (*P* < 0.01) treated groupsin terms of tail length (Figure 4(f)).

### 3.5. Preventive Effect of TCM on MPTP Induced Dopaminergic Neuron Loss in SNpc

**Figure 5:**
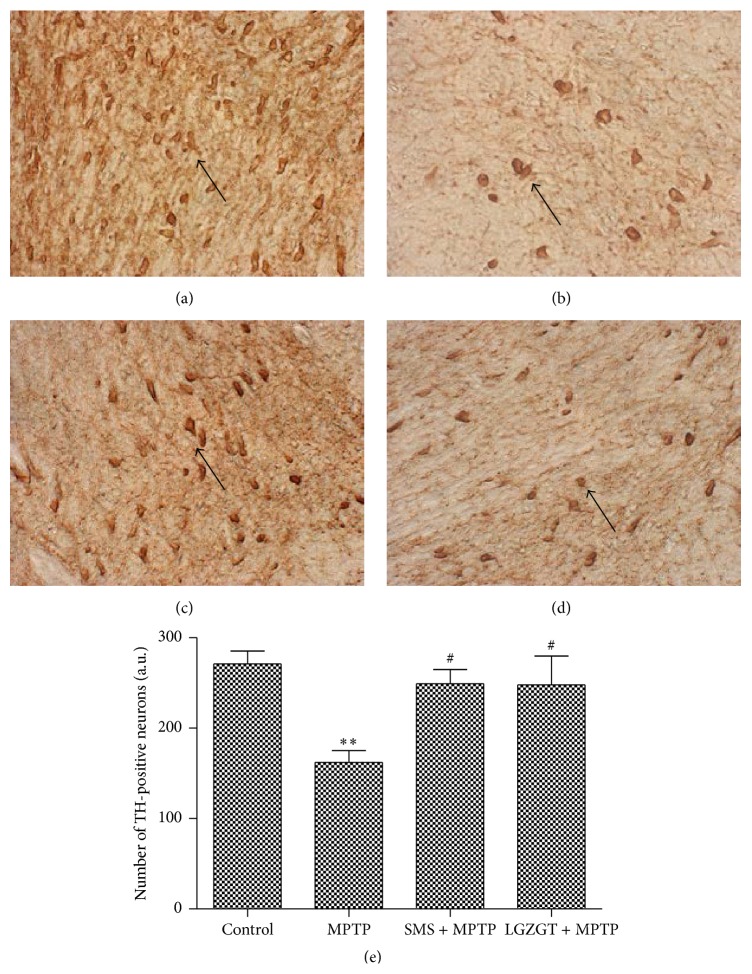
Preventive effect of SMS and LGZGT on MPTP induced dopaminergic neurons loss in SNpc. Dopaminergic neurons were immunohistochemically determined by the antibody reactive to tyrosine hydroxylase as described in the method section. (a) Control, (b) MPTP + saline, (c) SMS + MPTP, and (d) LGZGT + MPTP. Values are mean ± SE. *N* = 5, ^*∗∗*^ *P* < 0.01: significantly different from control group. ^#^ *P* < 0.05: significantly different from the MPTP only treated group.

The effects of SMS and LGZGT on the number of TH-immunoreactive dopaminergicneurons were examined immunohistochemically and the results are summarized in Figure 5, in order to rationale the preventive effect of TCM on the above observed behavioral dysfunction. On the day 11, 5 animals from each group have undergone immunohistochemical study after behavioral test. There was a significant(*P* < 0.01) loss of TH-immunoreactive neurons in the SNpc of MPTP treated mice, as compared to control (Figures 5(a) and 5(b)). The treatment with SMS (*P* < 0.05)and/or LGZGT significantly (*P* < 0.05)prevented the loss of dopaminergic neurons and the cell densities were comparable to those of control group (Figures 5(c) and 5(d)).

## 4. Discussion

In the present study, we examined the protective effect of two TCM formulae,SMS and LGZGT against MPTP induced neurotoxicity. Results from behavioral, biochemical, and immunohistochemical studies revealed that both SMS and/or LGZGT treatment were effective in attenuating MPTP induced neurotoxicity.

Extensive evidence suggest that protoxin MPTP is oxidized to 1-methyl-4-phenyl-2,3-dihydropyridinium (MDPD^+^) by monoamine oxidase B (MAO-B) after systemic administration. It is then converted to MPP^+^ which has a high affinity substrate for dopamine transporter and for norepinephrine and serotonin transporters [29, 30]. MPP^+^ also induces mitochondrial dysfunction with a pathologic cascade involving both excitotoxicity and free radical production. It was reported that the brains of mice treated with MPTP exhibited the decreased complex I activity, the increased hydroxyl radical production, and the elevated activities of superoxide dismutase (SOD), catalase, and glutathione peroxidase (GPx) [31, 32].

Indeed, there are reports that the levels of markers of oxidative damage to proteins, such as carbonyl modifications of soluble proteins, are significantly increased in postmortem samples of SNpc in PD [33, 34]. Therefore, the oxidative stress is accepted as the critical factor in pathogenesis of PD, and several antioxidants have been examined to protect against MPTP induced neurotoxicity including natural antioxidant like curcumin [3, 35, 36].

However, PD is a multifactorial disease involving oxidative/nitrative stress, mitochondrial dysfunction, protein aggregation, proteasome inhibition, and so forth. Most of these pathways are interlinked, but the molecular details of their relationships are still remained unclear [1, 4]. Moreover, neither the contribution nor the chronologies of all the pathways in neurodegeneration have been ascertained yet. Therefore, researchers have been screening for drugs that are therapeutically effective against most of these pathways. This emphasized the necessity of agents that are simultaneously effective against multiple pathways in PD [36].

In this sense, TCM prescriptions with antioxidant potential are attractive target of study as an alternative treating modality of cerebral degenerative diseases such as PD and Alzheimer's disease. They are basically antioxidant based multiple functional formula because they are comprised of several herbal constituents with different functional property, and many of herbs are usually antioxidant active that will be essential function for protecting neuronal cell against oxidative stress.

The TCM formulae, SMS, and LGZGT studied in the present experiments are used for different disease conditions. LGZGT has been frequently applied for the symptoms associated with brain functions such as dizziness and SMS clinically for the treatment of coronary heart diseases [14–16]. Moreover, both formulae had strong antioxidant activity as the IC_50_ values were comparable to reference antioxidants, Trolox, and BHT* in vitro*, but their scavenging properties were somewhat different. SMS was rather specific scavenger of hydroxyl radical, but LGZGT showed stronger activity towards superoxide and DPPH radicals* in vitro* (Table 1).

The neuroprotective function of SMS has been reported elsewhere, such that it protects rat brain against heart stroke-induced arterial hypotension and cerebral ischemia by inhibiting the prooxidant enzyme nitric oxide synthase [37]. In our earlier studies, SMS was shown to upregulate the antioxidant enzymes such as glutathione reductase, GPx, and SOD against scopolamine induced amnesia [18]. In the present study, not only SMS but also LGZGT with differential radical scavenging property prevented oxidative tissue damages in the brain (midbrain and cortex) of mice treated with MPTP as shown by the inhibitory action on formation of MDA and protein carbonyls (Figure 3). Further study on the DNA damage protection also indicated effective antioxidant property of both TCM formulae* in vivo* (Figure 4).

These antioxidant properties were finely reflected in their protective action on MPTP induced neurobehavioral impairments in MPTP model mice of PD used elsewhere [38, 39] as it is predicted from the previous reports that MPTP induced neurotoxicity associated with oxidative stress and DNA damage [40, 41]. MPTP treatment significantly decreased the locomotionand rearing frequencies and increased the latency to start the first movement and immobility time (Figures 1 and 2), but both TCM formulae significantly prevented the behavioral impairments induced by MPTP. Along with this observation, the MPTP induced loss of dopaminergic neurons in the SNpc was also prevented by SMS and LGZGT (Figure 5). Age related MPTP neurotoxicity has been hypothesized to be due to an increase of MPP^+^ concentration with age, because young mice excluded MPP^+^ rapidly than older ones [42]. In the present study, mice of age about 12–15 weeks were selected. The percentage reduction of TH neurons was 40% as compared to control; this is consistent with He et al. [42]. Further, there was not much observed difference in their protective effect on MPTP induced oxidative damages but for a slight difference in behavioral results between both formulae. These results suggest the multifunctional property of antioxidant formulae will be another factor in their protective function.

Currently, antioxidant compounds are widely recognized for the potential therapeutic treatment of diseases associated with oxidative stress. Several, antioxidant drugs, natural products, and antioxidant materials in foods have been tested in the MPTP mouse model [43, 44]. Sch B, a lignan isolated from* Schisandra chinensis* that is one of the herbal components of SMS, was also active in protecting oxidative damage and functional deficit of mice brain induced by cisplatin [45]. In contrast, TCM prescriptions are usually prescribed with multiple herbal components. Therefore, complex interactions, such as additive, synergistic, and antagonistic interactions are involved in the activities of the component herbs. These interactions are considered essential in improving their therapeutic potentiality and in reducing the side effects of certain toxic ingredients [46]. It is not yet understandable whether the mixtures are more advantageous than a single molecule. Therefore, further studies are required to clarify the underlying molecular mechanism of components interaction and develop an appropriate system to allow the evaluation of the preventive and/or ameliorative potential of such mixed formulae against complex diseases including PD.

## 5. Conclusion

The present study revealed that two traditional oriental formulae, Shengmai San and LingGuiZhuGanTang, covering different pathological applications, one is used for brain related disorder and the other is used for heart related disorder, with differential radical reactivity, effectively prevented MPTP induced cerebral oxidative stress and neurobehavioral impairments. Although further studies are required, the present observations suggest the potential use of these antioxidant oriental medicines to treat degenerative diseases in complementary and integrated medicine.

## Figures and Tables

**Table 1:** *In vitro* antioxidant activities of SMS and LGZGT.

Antioxidant assays	SMS IC_50_ (*μ*g/mL)	LGZGT IC_50_ (*μ*g/mL)	Trolox IC_50_ (*μ*g/mL)	BHT IC_50_ (*μ*g/mL)
Hydroxyl radical scavenging activity	45.6	98.6	18.9	34.3
Superoxide scavenging activity	298.6	103.6	100.0	98.4
DPPH scavenging activity	34.7	15.8	10.8	20.0

The antioxidant activities were determined as described in Section 2.
